# Long-Term Oncologic Outcome of Breast-Conserving Treatment in Patients With Breast Cancer With *BRCA *Variants

**DOI:** 10.1001/jamanetworkopen.2025.9840

**Published:** 2025-05-14

**Authors:** Janghee Lee, Jai Min Ryu, Hong Kyu Kim, Hyung Seok Park, Byeongju Kang, Sung Gwe Ahn, Min Sung Chung, Seon-Hi Shin, Junwon Go, Sanghwa Kim, Eun Young Kim, Young-Joon Kang, Sun Young Min, Moohyun Lee, Eunju Shin, Jisoo Shin, Sae Byul Lee, Chihwan David Cha

**Affiliations:** 1 Department of Surgery, Ewha Womans University Mokdong Hospital, Ewha Womans University College of Medicine, Seoul, Republic of Korea; 2 Department of Medicine, Yonsei University College of Medicine, Seoul, Republic of Korea; 3 Division of Breast Surgery, Department of Surgery, Sungkyunkwan University School of Medicine, Samsung Medical Center, Seoul, Republic of Korea; 4 Breast Care Center, Department of Surgery, Seoul National University Hospital, Seoul, Republic of Korea; 5 Department of Surgery, Yonsei University College of Medicine, Seoul, Republic of Korea; 6 Department of Surgery, School of Medicine, Kyungpook National University, Kyungpook National University Chilgok Hospital, Daegu, Republic of Korea; 7 Department of Surgery, Gangnam Severance Hospital, Yonsei University College of Medicine, Seoul, Republic of Korea; 8 Department of Surgery, Hanyang University Medical Center, Hanyang University College of Medicine, Seoul, Republic of Korea; 9 Biostatistical Consulting and Research Laboratory, Medical Research Collaborating Center, Hanyang University, Seoul, Republic of Korea; 10 Department of Radiology, New York University Grossman School of Medicine; 11 Department of Breast and Endocrine Surgery, Hallym University Sacred Heart Hospital, Hallym University, Anyang, Republic of Korea; 12 Department of Surgery, Kangbuk Samsung Hospital, Sungkyunkwan University School of Medicine, Seoul, Republic of Korea; 13 Department of Surgery, Incheon St Mary’s Hospital, College of Medicine, The Catholic University of Korea, Incheon, Republic of Korea; 14 Department of Surgery, Kyung Hee University Hospital, Seoul, Republic of Korea; 15 Department of Surgery, Keimyung University School of Medicine, Daegu, Republic of Korea; 16 Division of Breast Surgery, Department of Surgery, University of Ulsan College of Medicine, Asan Medical Center, Seoul, Republic of Korea

## Abstract

**Importance:**

Patients with sporadic breast cancer have comparable prognoses after undergoing either breast-conserving treatment (BCT) or mastectomy. However, there are limited and inconsistent data on the assessment of oncologic outcomes between BCT and mastectomy in patients with pathogenic variants in *BRCA1* or *BRCA2*.

**Objective:**

To investigate the outcomes of BCT on recurrence and survival in patients with breast cancer with *BRCA1 *or *BRCA2* pathogenic variants.

**Design, Setting, and Participants:**

This retrospective multicenter cohort study analyzed patients from 13 institutions in South Korea with primary breast cancer with *BRCA1 *or *BRCA2* pathogenic variants who underwent either BCT or mastectomy from January 2008 through December 2015. The median (IQR) follow-up period was 8.3 (6.4-9.6) years. Data were analyzed from September 2023 to August 2024.

**Exposure:**

*BRCA1 *or *BRCA2* pathogenic variant and BCT.

**Main Outcomes and Measures:**

Primary outcomes were logoregional recurrence–free survival, distant recurrence–free survival, and overall survival. Propensity score matching (PSM) using the greedy nearest neighbor method was performed to match covariates to minimize potential selection bias.

**Results:**

A total of 575 female patients with *BRCA1 *or *BRCA2* pathogenic variants were identified, all of whom were South Korean with a mean (SD) age of 42.0 (9.7) years. Among them, 367 patients (66.2%) received BCT and 186 (33.8%) were treated with mastectomy. BCT was not a factor associated with oncologic outcomes, including locoregional recurrence, compared with mastectomy. After adjusting for clinicopathologic characteristics through 1:1 PSM, there were still no statistically significant differences in oncologic outcomes between the BCT group and the mastectomy group. Multivariate analysis showed that the type of breast surgery was not significantly associated with oncologic outcomes. In subgroup analysis among matched patients based on *BRCA1 *or *BRCA2* status, tumor size, lymph node metastasis, histologic grade, and subtype, BCT was also not a factor associated with risk for recurrence.

**Conclusions and Relevance:**

The findings from this cohort study of patients with *BRCA1 *or *BRCA2* pathogenic variants suggested that there were no significant differences in oncologic outcomes between patients who underwent BCT and those who underwent mastectomy. Therefore, breast conservation with close surveillance can be considered a viable treatment option for *BRCA1 *or *BRCA2* pathogenic variant carriers. Further studies incorporating prospectively collected data are warranted to validate our findings.

## Key Points

**Question:**

Is breast-conserving treatment (BCT) comparable with mastectomy in terms of oncologic outcomes in patients with breast cancer with *BRCA1* or *BRCA2* pathogenic variants?

**Findings:**

In this cohort study of 575 patients using propensity score matching, among patients with breast cancer with *BRCA1 *or *BRCA2* pathogenic variants, there was no significant difference in oncologic outcomes such as locoregional recurrence, distant recurrence, and overall survival between BCT group and mastectomy group during a median follow-up of 8.3 years.

**Meaning:**

These findings suggest BCT can be considered a viable treatment option for patients with breast cancer with *BRCA1 *or* BRCA2* pathogenic variants since it is regarded as safe compared with mastectomy.

## Teaser

This cohort study evaluates the long-term outcomes of breast-conserving treatment (BCT) on recurrence and survival in patients with breast cancer with *BRCA1* and *BRCA2* pathogenic variants.

## Introduction

Breast cancer remains the most prevalent malignant neoplasm in women in most countries, with an annual increase in incidence of 2%.^1,2^ Among numerous risk factors for breast cancer, pathogenic variants in *BRCA1* and *BRCA2* have gained significant attention due to their genetic characteristics and potential impact on treatment decisions.^3^ *BRCA1* and *BRCA2* are distinct tumor suppressor genes that play an integral role in responding to cellular stress through the activation of DNA repair processes.^3,4^ Individuals with pathogenic variants in *BRCA1* or *BRCA2* face an elevated risk of developing breast cancer, with lifetime risk ranging from 20% to 65%.^5,6,7,8,9^

Breast-conserving treatment (breast-conserving surgery plus radiation, BCT) has long been established as a viable alternative to mastectomy for patients with breast cancer. Many previous studies have demonstrated that BCT is not inferior to mastectomy and yields superior cosmetic outcomes.^10,11,12,13,14,15^ However, the suitability and safety of BCT in patients with *BRCA1 *or *BRCA2* pathogenic variants remain relatively uncertain. Several studies have investigated the oncologic safety of BCT in patients with *BRCA* pathogenic variants, but the results have varied among these studies.^16,17,18,19,20,21,22,23,24^ Therefore, current guidelines still specify that patients with breast cancer with genetic predispositions, such as *BRCA1 *or *BRCA2* pathogenic variants, may consider prophylactic bilateral mastectomy for risk reduction.^25^

The purpose of our study is to assess the oncologic safety of BCT in patients carrying *BRCA1 *or *BRCA2* pathogenic variants by comparing long-term outcomes with mastectomy. Our assessment includes not only distant recurrence (DR) and overall survival (OS) but also locoregional recurrence (LRR). By providing insights into the oncologic outcomes of BCT for patients with *BRCA1 *or *BRCA2* pathogenic variants, we aim to present evidence to aid in surgical decision-making for the care of these patients.

## Methods

### Study Populations

This study followed the Strengthening the Reporting of Observational Studies in Epidemiology (STROBE) reporting guideline for observational studies. Our study adhered to Good Clinical Practice guidelines and the principles of the Declaration of Helsinki. This study was approved by the institutional review board of Hanyang University Hospital. The retrospective study design warranted a waiver for the requirement of written informed consent by the institutional review board. The study was conducted in accordance with Strengthening The Reporting Of Cohort Studies in Surgery (STROCSS) criteria.^26^ The ON-BRCA II is a multi-institution cohort study being conducted by the Korea Robot-Endoscopy Minimal Access Breast Surgery Study Group. Patients with primary breast cancer who underwent BCT or mastectomy and received *BRCA1 *or *BRCA2* variant testing between January 2008 and December 2015 were retrospectively identified from 13 institutions in South Korea. Inclusion criteria were patients aged 20 to 80 years with invasive breast cancer (pT1-3 or N0-3). Exclusion criteria were patients with de novo metastasis and those with pregnancy-associated breast cancer.

All enrolled patients underwent mammogram, breast ultrasound, and magnetic resonance imaging (MRI) before treatment, and operational details, including the date of operation and the type of breast and axillary surgery, were recorded. Clinicopathologic characteristics were also collected using medical record review. We documented any additional treatments received, including chemotherapy, hormone therapy, target therapy, and radiation therapy. All instances of breast cancer recurrence, including LRR, DR, and death, were recorded during the follow-up period. Additionally, we investigated the prevalence of contralateral breast cancer (CBC) events in our cohort. The results of *BRCA1 *or *BRCA2* pathogenic variant tests were collected for all patients.

### *BRCA1 *or *BRCA2* Variant Testing

The screening of *BRCA1 *or *BRCA2* pathogenic variants was performed by analyzing genomic DNA extracted from the peripheral blood of patients. The coding regions and exon or intron boundaries of the *BRCA1 *or *BRCA2* genes were amplified using polymerase chain reaction. All deleterious variants were confirmed through Sanger sequencing in duplicate. Pathogenic variants were defined as those that lead to a truncated protein or have been reported previously as disease-associated.

### Statistical Analyses

The clinicopathological characteristics between the 2 groups were compared using *t* tests and χ^2^ tests. To mitigate potential selection bias due to the retrospective nature of the study, propensity score matching (PSM) with the greedy nearest neighbor matching method was performed for all covariates in each group to adjust for confounding factors. The covariates included for computing propensity scores were age, tumor size, lymph node (LN) metastasis, histologic grade, hormone receptor (HR) or ERBB2 status, and chemotherapy. Each treated unit was matched with 1 control unit, resulting in a 1:1 greedy nearest neighbor matching method. After matching the propensity scores, χ^2^ tests, Fisher exact tests, and *t* tests were performed to assess the balance between the 2 groups. The caliper was set to 0.1, maintaining the absolute value of the difference in logits of propensity scores at 0.1 or below. Additionally, we validated our findings through analysis of patients matched using the inverse probability of treatment weighting (IPTW) method. A 2-sided *P *value less than .05 was considered significant.

The primary outcomes of interest in this study were LRR-free survival (LRRFS), DR-free survival (DRFS), recurrence-free survival (RFS), and OS after breast-conserving surgery or mastectomy in patients carrying *BRCA1 *or *BRCA2* pathogenic variants. LRRFS was defined as the duration from diagnosis until the development of recurrence in breast or chest wall and/or regional LNs on the side previously affected by cancer. DRFS was defined as the duration from diagnosis until the development of recurrence in a distant organ. RFS was defined as the duration from diagnosis until any form of disease recurrence was detected. OS was defined as the duration from diagnosis until death. Since our study is a retrospective design, we did not separately exclude patients lost to follow-up. If there was no recurrence or death event during follow-up period, the patient was considered event-free.

The Kaplan-Meier method was used to estimate the prognosis in the 2 groups. The log-rank test was used to compare survival outcomes according to the type of surgery. We conducted multivariate analysis using Cox proportional hazard regression, adjusting for covariates such as age, tumor size, LN metastasis, and tumor subtype, which are known to be associated with breast cancer prognosis. Additionally, we calculated hazard ratios for survival outcomes along with 95% CIs. All analyses were performed using SAS version 9.4 (SAS Institute Inc) and SPSS version 27 (IBM). Data were analyzed from September 2023 to August 2024.

## Results

### Patient Characteristics

**Figure 1.**
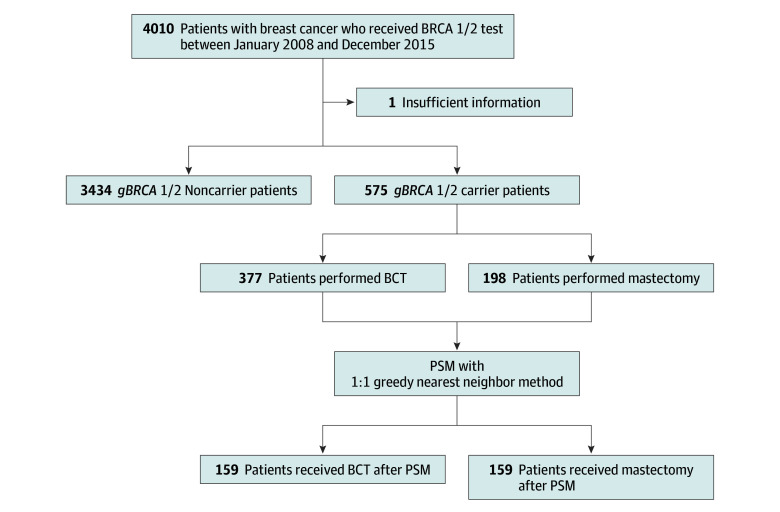
Diagram of Enrolled Patients BCT indicates breast-conserving treatment; PSM, propensity score matching.

We retrospectively collected information on 4010 female patients with primary breast cancer who underwent *BRCA* genetic testing followed by curative surgery. From this group, we selected 575 patients (14.3%) who had pathogenic *BRCA* pathogenic variants, all of whom were South Korean (Figure 1). The mean (SD) age was 42.0 (9.7) years, and 251 patients (43.7%) had triple-negative breast cancer (TNBC). Among these patients, 377 (65.6%) received BCT, while 198 (34.4%) underwent mastectomy. Of the 575 patients, 338 (58.8%) had a *BRCA1* pathogenic variant, 223 (38.8%) had a *BRCA2* pathogenic variant, and 14 (2.4%) had both pathogenic variants. More than half of the patients had a high histological grade (HG), and about 80% received chemotherapy. In cases where prophylactic surgeries were performed concurrently, 46 patients (8.3%) underwent contralateral mastectomy, and 135 (24.4%) underwent bilateral salpingo-oophorectomy (BSO). eTable 1 in Supplement 1 summarizes the baseline characteristics according to the type of breast surgery.

We conducted PSM to address the significant differences in several clinicopathologic factors between the group that underwent BCT and mastectomy. After performing 1:1 PSM using the greedy-nearest-neighbor method to adjust for age, tumor size, LN metastasis, HG, and subtype, the variables between the 2 groups were well balanced (Table 1). The standardized mean difference of the logit propensity score was 0.017, which is well below the upper limit of 0.10. Each group included 159 patients (Figure 1).

**Table 1.** Comparison of Clinicopathologic Factors Based on Breast Surgery Type Before and After 1:1 Propensity Score Matching

Variable	Unmatched			Matched		
	Patients, No. (%)		*P* value	Patients, No. (%)		*P* value
	BCT (n = 377)	Mastectomy (n = 198)		BCT (n = 159)	Mastectomy (n = 159)	
Age, mean (SD), y	41.8 (9.4)	42.3 (10.0)	.54	42.5 (9.6)	43.0 (10.2)	.71
*BRCA*						
* BRCA1*	237 (62.9)	101 (51.0)	.004	87 (54.7)	96 (60.4)	.34
* BRCA2*	135 (35.8)	88 (44.5)		72 (45.3)	62 (39.0)	
*BRCA1* and *BRCA2*	5 (1.3)	9 (4.5)		0	1 (0.6)	
Risk-reducing BSO						
Not performed	269 (71.4)	160 (80.8)	.01	114 (71.7)	127 (79.9)	.09
Performed	108 (28.6)	38 (19.2)		45 (28.3)	32 (20.1)	
Tumor size (mm)						
≤20	252 (66.8)	99 (50.0)	<.001	92 (57.9)	87 (54.7)	.65
>20	120 (31.8)	98 (49.5)		67 (42.1)	71 (44.7)	
Unknown	5 (1.3)	1 (0.5)		0	1 (0.6)	
Lymph node metastasis						
Negative	281 (74.5)	106 (53.5)	<.001	98 (61.6)	100 (62.9)	.82
Positive	92 (24.4)	91 (46.0)		61 (38.4)	58 (36.5)	
Unknown	4 (1.1)	1 (0.5)		0	1 (0.6)	
Histologic grade						
I/II	128 (34.0)	103 (52.0)	<.001	72 (45.3)	71 (44.7)	.97
III	224 (59.4)	83 (41.9)		78 (49.1)	78 (49.1)	
Unknown	25 (6.6)	12 (6.1)		9 (5.7)	10 (6.3)	
Subtype						
HR+/ERBB2−	163 (43.2)	111 (56.1)	.004	79 (49.7)	82 (51.6)	.95
ERBB2+	24 (6.4)	16 (8.1)		14 (8.8)	14 (8.8)	
TNBC	185 (49.1)	66 (33.3)		63 (39.6)	59 (37.1)	
Unknown	5 (1.3)	5 (2.5)		3 (1.9)	4 (2.5)	
CTx						
Not performed	86 (22.8)	35 (17.7)	.33	34 (21.4)	31 (19.5)	.92
Performed	290 (76.9)	162 (81.8)		124 (78.0)	127 (79.9)	
Unknown	1 (0.3)	1 (0.5)		1 (0.6)	1 (0.6)	

Abbreviations: +, positive; −, negative; BCT, breast-conserving treatment; BSO, bilateral salpingo-oophorectomy; CTx, chemotherapy; ERBB2, human epidermal growth factor receptor 2; HR, hormone receptor; TNBC, triple negative breast cancer.

### Recurrence, Death, and CBC Events in Entire Cohort

The 5-year RFS and OS rates were 91.1% and 96.6%, respectively. During median (IQR) follow-up of 8.3 (6.4-9.6) years, there were 184 cases of recurrence, death, or contralateral breast cancer events (eTable 2 in Supplement 1). The rates of LRR and DR were 4.9% and 9.0%, respectively, and 35 patients (6.3%) died during the follow-up period. Among patients who did not undergo prophylactic contralateral mastectomy, 72 (14.2%) experienced metachronous contralateral breast cancer (MCBC).

### Survival Analysis According to Breast Surgery Type

In the multivariable analysis of enrolled patients, the type of breast surgery was not a significant factor for oncologic outcomes, including locoregional recurrence (LRRFS: hazard ratio [HR], 1.87; 95% CI, 0.79-4.42; *P* = .16; DRFS: HR, 1.36; 95% CI, 0.76-2.48; *P* = .29; RFS: HR, 1.47; 95% CI, 0.93-2.33; *P* = .10; OS: HR; 1.03; 95% CI, 0.50-2.13; *P* = .94) (eTable 3 in Supplement 1). Tumor size, the performance of risk-reducing BSO, and LN metastasis were factors associated with patient prognosis.

**Figure 2.**
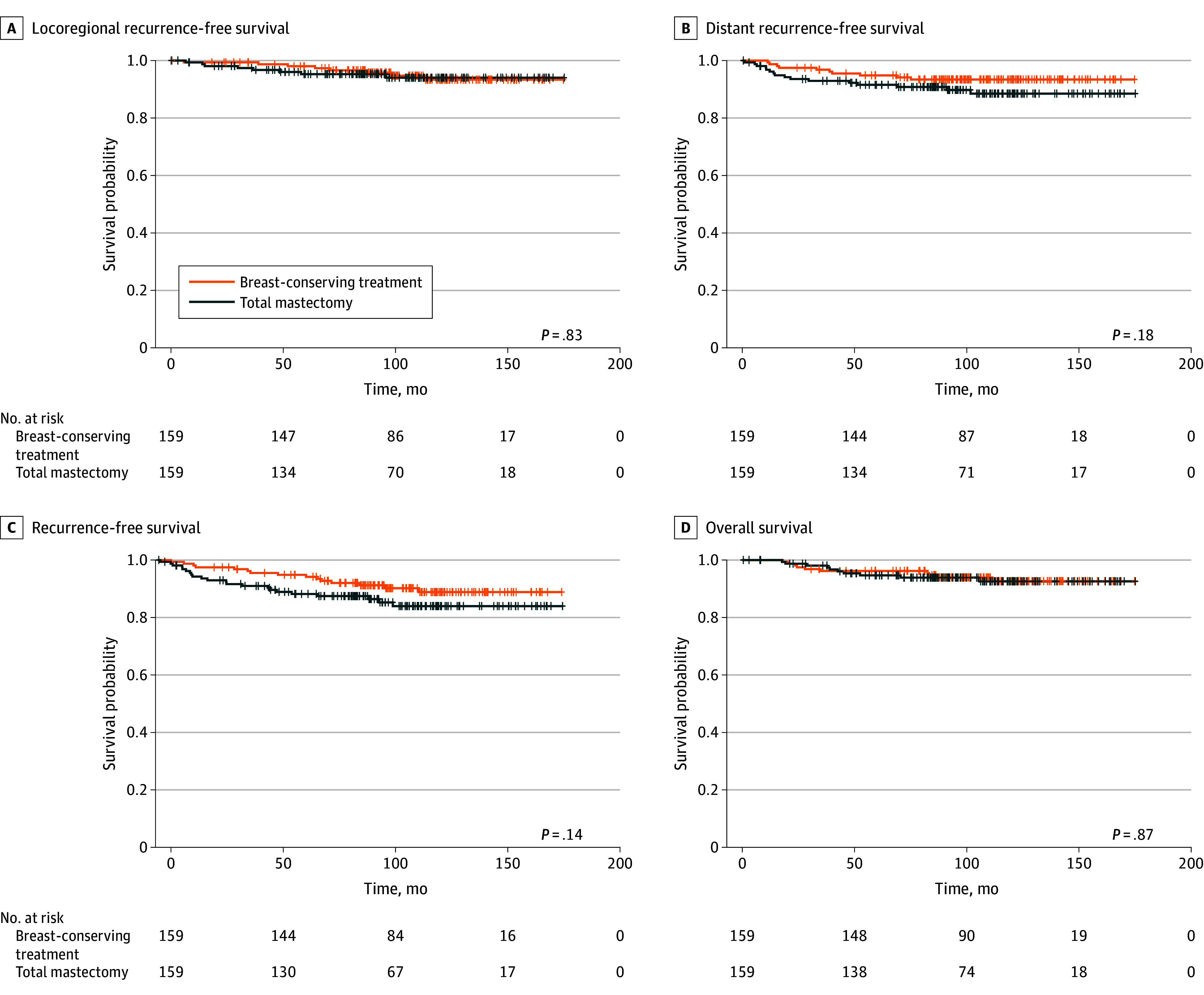
Kaplan-Meier Survival Curve of Breast Surgery Type in 1:1 Propensity Score Matched Patients

In Kaplan-Meier survival curves, no significant difference was observed in recurrence and survival outcomes between patients who received BCT and those who underwent mastectomy, selected through 1:1 PSM (Figure 2). LRRFS did not show a difference between the 2 groups. While DRFS and RFS appeared slightly superior in the BCT group, it was not statistically significant. Additionally, there was no difference in OS. In the multivariate analysis with 309 patients, representing a loss of about 2.8% of participants, breast surgery type was not a statistically significant factor associated with survival outcomes (LRRFS: HR, 0.96; 95% CI, 0.36-2.59; DRFS: HR, 0.62; 95% CI, 0.28-1.38; RFS: HR, 0.63; 95% CI, 0.33-1.22; OS: HR, 0.82, 95% CI, 0.34-1.98) (Table 2). Tumor size was the sole factor associated with risk for DRFS, and the presence of LN metastasis was a significant poor factor for OS (tumor size for DRFS: HR, 3.87; 95% CI, 1.51-9.94; *P* < .01; LN metastasis for OS: HR, 3.78; 95% CI, 1.44-9.97; *P* < .01). In additional analysis of patients matched using the IPTW method, there was no significant difference in oncologic outcomes between BCT and mastectomy (eFigure in Supplement 1).

**Table 2.** Multivariate Analysis of Breast Surgery Type for Survival Outcomes in 1:1 Propensity Score–Matched Patients

Variable (reference category)	Hazard ratio (95% CI) of each model			
	LRRFS	DRFS	RFS	OS
Surgery type (mastectomy)				
BCT^a^	0.90 (0.34-2.39)	0.59 (0.27-1.29)	0.62 (0.32-1.19)	0.93 (0.39-2.23)
BCT^b^	0.96 (0.36-2.59)	0.62 (0.28-1.38)	0.63 (0.33-1.22)	0.82 (0.34-1.98)
Age	0.96 (0.91-1.02)	1.01 (0.97-1.05)	1.00 (0.97-1.04)	0.94 (0.88-0.99)^c^
Tumor size (≤20 mm)				
>20 mm	1.20 (0.43-3.32)	3.87 (1.51-9.94)^d^	2.19 (1.10-4.37)^c^	1.69 (0.64-4.42)
Lymph node metastasis (negative)				
Positive	0.23 (0.05-1.02)	1.77 (0.80-3.90)	0.96 (0.48-1.90)	3.78 (1.44-9.97)^d^
Histologic grade (I/II)^e^				
III	0.70 (0.21-2.29)	0.85 (0.32-2.29)	0.97 (0.44-2.17)	1.33 (0.38-4.69)
Subtype (HR+/ERBB2−)				
ERBB2+	1.48 (0.30-7.39)	0.52 (0.07-4.15)	0.94 (0.27-3.29)	0.70 (0.08-6.23)
TNBC	1.01 (0.31-3.28)	1.48 (0.54-4.11)	1.10 (0.49-2.46)	2.00 (0.60-6.62)

Abbreviations: +, positive; −, negative; BCT, breast-conserving treatment; DRFS, distant recurrence-free survival; ERBB2, human epidermal growth factor receptor 2; HR, hormone receptor; LRRFS, locoregional recurrence-free survival; OS, overall survival; RFS, recurrence-free survival; TNBC, triple negative breast cancer.

^a^ Statistics were obtained from the unadjusted model.

^b^ Statistics were obtained from the adjusted model.

^c^ *P* ≤ .05.

^d^ *P* < .01.

^e^ The unknown responses for each variable except histologic grade were considered missing. The missingness rate of the analyses was less than 5%. The category of missing responses of histologic grade is omitted in this table due to lack of interpretability.

For the incidence of survival events, there were 8 patients with LRR in both subgroups (eTable 4 in Supplement 1). The number of patients with DR was 10 (6.4%) in the BCT group, and 16 (10.3%) in mastectomy group. Regarding the events of MCBC, defined as diagnosed more than 1 year following the surgery of the primary cancer, there was no difference between the 2 groups (13.3% vs 10.7%). Similarly, the incidence of OS events was not significantly different.

**Figure 3.**
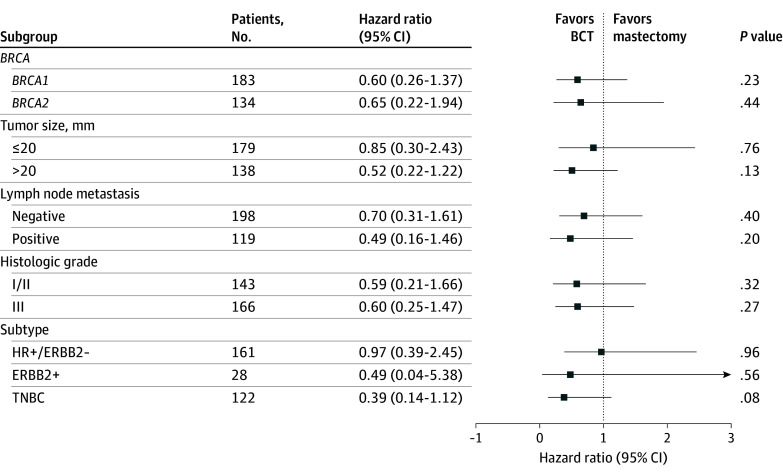
Forest Plot for Subgroup Analysis in 1:1 Propensity Score Matched Patients + indicates positive; −, negative; BCT, breast-conserving treatment; ERBB2, human epidermal growth factor receptor 2; HR, hormone receptor; TNBC, triple negative breast cancer.

Finally, we performed additional subgroup analysis on patients matched 1:1 using PS based on tumor size, LN metastasis, HG, and subtype (Figure 3). In all subgroups, including each group with *BRCA1 *or *BRCA2* pathogenic variants, the type of breast surgery did not emerge as a significant factor associated with risk for recurrence.

## Discussion

Our large-scale retrospective multicenter study found that BCT is comparable with mastectomy in terms of oncologic outcomes for patients with breast cancer who carry *BRCA1 *or *BRCA2* pathogenic variants. Furthermore, we found no significant differences not only in DRFS and RFS but also in LRRFS. Our results suggest that BCT can be considered a safe treatment option for patients with breast cancer carrying *BRCA1 *or *BRCA2* pathogenic variants.

*BRCA1 *or *BRCA2* are well-known as DNA repair genes, and it is widely recognized that the incidence of breast cancer significantly increases in the presence of pathogenic variants in these genes.^27,28,29^ According to The Cancer Genome Atlas network, germline *BRCA1 *or *BRCA2* pathogenic variant carriers account for 3% to 4% of all patients with breast cancer.^30^ However, previous clinical studies have reported that the proportion of *BRCA* pathogenic variant carriers is around 10% to 20%.^31,32^ In our cohort, the prevalence of *BRCA* pathogenic variants was about 13.8% (553 of 4010), which is consistent with the results of previous clinical studies. These results likely stem from the selective identification of patients with a higher probability of *BRCA* pathogenic variants, such as young age or TNBC, through genetic counselling in clinical settings.

It is well-documented that the incidence of CBC is higher in individuals with *BRCA* pathogenic variants compared with those without.^33,34^ Sun et al^35^ have reported that approximately 13.4% of patients with *BRCA* pathogenic variants experience CBC, while Su et al^36^ have found that the 10-year cumulative risk of CBC in Chinese patients with breast cancer with *BRCA* pathogenic variants ranges from 15.5% to 17.5%. In our cohort, the incidence of CBC in individuals with *BRCA* pathogenic variants was 14.2%, which is consistent with previous studies and higher than the proportion observed in noncarrier patients. Notably, the incidence rate of CBC did not significantly differ between the BCT and mastectomy groups, suggesting that intensive surveillance following BCT may offer a viable alternative to prophylactic mastectomy for select patients.

Moreover, new primary breast cancers may arise in *BRCA1 *or *BRCA*2 variant carriers due to inherent genetic susceptibility. While LRR has traditionally been a key metric in oncologic safety assessments, for *BRCA* carriers, the risk of new primary tumors may be of equal or greater importance. However, unfortunately, our data did not include the occurrence of new primary cancers as an outcome measure. Further studies with the additional outcomes are warranted to investigate the long term risk of new primary events.

In our cohort, a significant portion of patients (24.4%) also underwent risk-reducing BSO. Given that BSO induces premature menopause by eliminating ovarian hormone production, it has critical implications for oncologic outcomes, especially in HR-positive patients. While our study did not specifically stratify outcomes based on the BSO status, its potential influence on survival outcomes must be considered when interpreting the results. Future analyses should explore the differential impact of BSO in *BRCA1* vs *BRCA2* carriers and its interaction with systemic therapies.

Our study confirmed that in patients with *BRCA1 *or *BRCA2* pathogenic variants, BCT is a safe surgical option in long-term oncologic outcomes including overall recurrence and DR compared with mastectomy. Most studies conducted among patients with breast cancer with *BRCA1 *or *BRCA2* pathogenic variants report no significant difference in DRFS, breast cancer-specific survival (BCSS), and OS when comparing BCT with mastectomy.^17,20,21,22^ Additionally, van den Broek et al^23^ have reported equivalent survival rates between BCT and mastectomy when specifically analyzing patients with breast cancer with *BRCA2* variants. Based on these results, guidelines recommend BCT for patients with breast cancer with *BRCA1 *or *BRCA2* pathogenic variants as moderate-level evidence.^25^ In the most recent study conducted on patients with *BRCA1* variants, although it was a univariate analysis, patients who underwent BCT demonstrated superior BCSS compared with those who underwent mastectomy.^37^ Our results provide stronger evidence for the eligibility for BCT in these patients with regard to DR and OS, based on a large-scale, long-term follow-up cohort utilizing advanced statistical methods such as PSM.

Concerns regarding ipsilateral breast tumor recurrence (IBTR) and regional recurrence in patients with breast cancer with *BRCA1 *or *BRCA2* pathogenic variants undergoing BCT still persist. Previous studies have reported conflicting results regarding LRR based on the type of breast surgery in these patients.^20,21,22,23,24^ Wanis et al^19^ recently analyzed *BRCA*-associated patients with breast cancer who underwent BCT in a single-institution study and reported that these patients had an above-average risk of IBTR and CBC events. Their findings support BCT as a safe survival option for patients with pathogenic *BRCA* variants. However, despite examining patients over a very long period from 1977 to 2021, the study included a relatively small sample size of only 172 patients and did not directly compare patients who underwent mastectomy with those who underwent BCT. On the other hand, in another current study, Nara et al^18^ argued through a meta-analysis that *BRCA* pathogenic carrier patients who underwent BCS with radiotherapy had a higher risk of IBTR compared with patients with sporadic breast cancer. However, their analysis failed to adjust for clinicopathologic characteristics of the patients, including age and stage, and did not compare the LRR of mastectomy and BCT in patients with *BRCA* pathogenic variants. Ultimately, our study confirmed that even in terms of LRR in *BRCA* carrier patients, BCT is a surgical option that is comparable with mastectomy in a multi-institutional large-scale cohort with long-term follow up. We believe that our forthcoming study, comparing *BRCA* pathogenic variant carriers with noncarriers, will further elucidate the safety of BCT.

### Strengths and Limitations

Our study has strength in the substantial collection of long-term follow-up data from a significant number of *BRCA* pathogenic variant carriers across 13 institutions. Furthermore, we made efforts to perform the most accurate analysis by adjusting several clinicopathologic features between the BCT group and mastectomy group through PSM. All patients in our study underwent preoperative breast MRI to accurately confirm eligibility for BCT, which guided the decision-making process for surgical approach.

Additionally, all patients in our cohort are of Asian descent, specifically Korean. Studying a homogeneous Asian population is significant, as genetic and environmental factors may differ from those of Western populations. This is particularly relevant given the limited availability of long-term follow-up data from large-scale studies focused exclusively on Asian populations. While our findings offer insights into the oncologic safety of BCT in this cohort, further research is needed to confirm these results across more diverse populations.

It is crucial to acknowledge some limitations associated with our study, primarily stemming from its retrospective nature. The potential for selection bias inherent in retrospective study designs cannot be entirely ruled out. In addition, our cohort does not clearly indicate whether the results of the BRCA pathogenic variant test were available before surgery. Since the presence of a BRCA pathogenic variant is an important factor in determining the surgical approach, biases may arise depending on when the results were reported. However, to minimize these biases, we utilized PSM and multivariate analysis. Furthermore, we were unable to include information on the precise site of *BRCA1 *or *BRCA2* pathogenic variants and other pathogenic variants, such as *TP53*, which could impact recurrence and prognosis in our analysis. Additional research in the future will be necessary to address these aspects.

## Conclusions

Our findings suggest that there was no difference in oncologic outcomes, including LRRFS, between *BRCA* pathogenic variant carriers who underwent BCT and those who underwent mastectomy. Therefore, breast conservation with close surveillance can be considered a reasonable treatment option for *BRCA* pathogenic variant carriers. However, further studies incorporating prospectively collected data are warranted to validate our findings.

## Notes

Supplement 1. **eFigure.** Kaplan-Meier survival curve of breast surgery type for prognosis after IPTW **eTable 1.** Baseline characteristics of enrolled patients with BRCA pathogenic variants **eTable 2.** Recurrence, death, and contralateral breast cancer events **eTable 3.** Multivariate analysis for survival outcomes in enrolled patients **eTable 4.** Incidence of survival events according to surgery type in 1:1 PS matched patients

Supplement 2.
Data Sharing Statement
