# Supplementary material for: Long-Term Oncologic Outcome of Breast-Conserving Treatment in Patients With Breast Cancer With BRCA Variants
Source: JAMA Netw Open. 2025 May 14;8(5):e259840. doi: 10.1001/jamanetworkopen.2025.9840 (PMC12079291; doi:10.1001/jamanetworkopen.2025.9840)
Supplement: Supplement 1. — eFigure. Kaplan-Meier survival curve of breast surgery type for prognosis after IPTW eTable 1. Baseline characteristics of enrolled patients with BRCA pathogenic variants eTable 2. Recurrence, death, and contralateral breast cancer events eTable 3. Multivariate analysis for survival outcomes in enrolled patients eTable 4. Incidence of survival events according to surgery type in 1:1 PS matched patients [file jamanetwopen-e259840-s001.pdf]

Supplemental Online Content

Lee J, Ryu JM, Kim HK. Long-term oncologic outcome of breast-conserving treatment in breast cancer with BRCA variants. *JAMA Netw Open*. 2025;8(5):e259840. doi:10.1001/jamanetworkopen.2025.9840

eFigure. Kaplan-Meier survival curve of breast surgery type for prognosis after IPTW

eTable 1. Baseline characteristics of enrolled patients with BRCA pathogenic variants

eTable 2. Recurrence, death, and contralateral breast cancer events

eTable 3. Multivariate analysis for survival outcomes in enrolled patients

eTable 4. Incidence of survival events according to surgery type in 1:1 PS matched patients

This supplemental material has been provided by the authors to give readers additional information about their work.

© 2025 Lee J et al. *JAMA Network Open*.

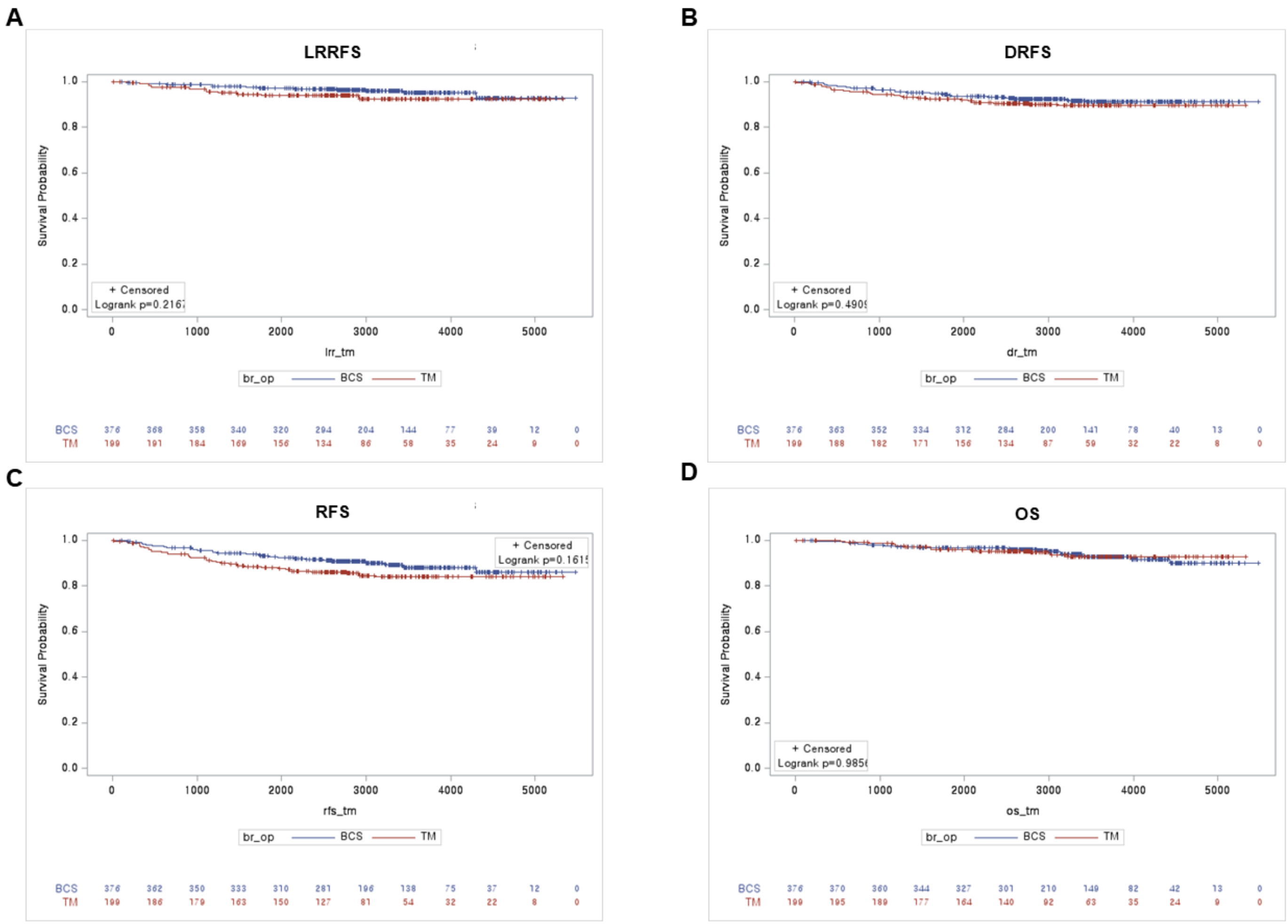

eFigure. Kaplan-Meier survival curve of breast surgery type for prognosis after IPTW. (A) LRRFS (*p* = .216). (B) DRFS (*p* = .49). (C) RFS (*p* = .615). (D) OS (*p* = .985).

BCS, breast-conserving surgery; TM, total mastectomy; LRRFS, locoregional recurrence-free survival; DRFS, distant recurrence-free survival; RFS, recurrence-free survival; OS, overall survival; IPTW, inverse probability of treatment weighting;

© 2025 Lee J et al. *JAMA Network Open*.

eTable 1. Baseline characteristics of enrolled patients with *BRCA* pathogenic variants

Variables	All patients, % (n=575)	Patients with BCT, % (n=377)	Patients with mastectomy, % (n=198)	*p*
Age (years), mean (SDs)	42.0 (9.6)	41.8 (9.4)	42.3 (10.0)	.54
BRCA				.004
BRCA1 positive	338 (58.8)	237 (62.9)	101 (51.0)	
BRCA2 positive	223 (38.8)	135 (35.8)	88 (44.5)	
BRCA1/2 positive	14 (2.4)	5 (1.3)	9 (4.5)	
Prophylactic contralateral mastectomy				<.001
Not performed	548 (95.3)	371 (98.4)	177 (89.4)	
Performed	27 (4.7)	6 (1.6)	21 (10.6)	
Risk-reducing BSO				.01
Not performed	429 (74.6)	269 (71.4)	160 (80.8)	
Performed	146 (25.4)	108 (28.6)	38 (19.2)	
Tumor size (mm)				<.001
≤20	351 (61.0)	252 (66.8)	99 (50.0)	
>20	218 (37.9)	120 (31.8)	98 (49.5)	
Unknown	6 (1.0)	5 (1.3)	1 (0.5)	
LN metastasis				<.001
Negative	387 (67.3)	281 (74.5)	106 (53.5)	
Positive	183 (31.8)	92 (24.4)	91 (46.0)	
Unknown	5 (0.9)	4 (1.1)	1 (0.5)	
HG				<.001
I/II	231 (40.2)	128 (34.0)	103 (52.0)	
III	307 (53.4)	224 (59.4)	83 (41.9)	
Unknown	37 (6.4)	25 (6.6)	12 (6.1)	

© 2025 Lee J et al. *JAMA Network Open*.

Subtype				.004
HR+/HER2-	274 (47.7)	163 (43.2)	111 (56.1)	
HER2+	40 (7.0)	24 (6.4)	16 (8.1)	
TNBC	251 (43.7)	185 (49.1)	66 (33.3)	
Unknown	10 (1.7)	5 (1.3)	5 (2.5)	
CTx				.33
Not performed	121 (21.0)	86 (22.8)	35 (17.7)	
Performed	452 (78.6)	290 (76.9)	162 (81.8)	
Unknown	2 (0.3)	1 (0.3)	1 (0.5)	

SD, standard deviation; BCT, breast-conserving treatment; BSO, bilateral salpingo-oophorectomy; LN, lymph node; HG, histologic grade; HR, hormone receptor; HER2, human epidermal growth factor receptor 2; TNBC, triple negative breast cancer; CTx, chemotherapy

© 2025 Lee J et al. *JAMA Network Open*.

eTable 2. Recurrence, death, and contralateral breast cancer events

Event	Patients (%)
Total events	184
Locoregional recurrence	27 (4.9)
Distant recurrence	50 (9.0)
Contralateral breast cancer among patients who not performed prophylactic contralateral mastectomy	72 (14.2)
Death	35 (6.3)

© 2025 Lee J et al. *JAMA Network Open*.

eTable 3. Multivariate analysis for survival outcomes in enrolled patients

Variables	LRRFS		DRFS		RFS		OS	
	Hazard ratio (95% CI)	*p*	Hazard ratio (95% CI)	*p*	Hazard ratio (95% CI)	*p*	Hazard ratio (95% CI)	*p*
Breast surgery								
BCT	Ref.		Ref.		Ref.		Ref.	
Mastectomy	1.87 (0.79-4.42)	.16	1.36 (0.76-2.48)	.29	1.47 (0.93-2.33)	.10	1.03 (0.50-2.13)	.94
Age (years), mean (SDs)	0.96 (0.91-1.01)	.08	1.01 (0.98-1.04)	.53	1.00 (0.98-1.02)	.90	0.98 (0.95-1.02)	.38
BRCA								
BRCA1 positive	Ref.		Ref.		Ref.		Ref.	
BRCA2 positive	0.99 (0.35-2.82)	.98	0.52 (0.26-1.05)	.07	0.83 (0.48-1.42)	.49	0.99 (0.41-2.40)	.97
BRCA1/2 positive	0 (0-NE)	.99	0.65 (0.15-2.81)	.56	0.75 (0.23-2.47)	.64	0.71 (0.09-5.53)	.74
Prophylactic contralateral mastectomy								
Not performed	Ref.		Ref.		Ref.		Ref.	
Performed	0 (0-NE)	.99	0.41 (0.06-3.02)	.38	0.25 (0.03-1.83)	.17	0 (0-NE)	.97
Risk-reducing BSO								
Not performed	Ref.		Ref.		Ref.		Ref.	
Performed	0.68 (0.23-2.05)	.49	0.47 (0.21-1.05)	.07	0.41 (0.22-0.79)	.007	0.17 (0.04-0.71)	.02
Tumor size (mm)								
≤20	Ref.		Ref.		Ref.		Ref.	
>20	1.94 (0.82-4.62)	.13	2.81 (1.55-5.12)	.001	2.46 (1.56-3.89)	<.001	2.03 (1.00-4.12)	.05
LN metastasis								
Negative	Ref.		Ref.		Ref.		Ref.	
Positive	0.83 (0.32-2.17)	.71	1.60 (0.88-2.92)	.13	1.33 (0.83-2.13)	.24	2.01 (1.01-4.30)	.047
HG								
I/II	Ref.		Ref.		Ref.		Ref.	

© 2025 Lee J et al. *JAMA Network Open*.

III	0.59 (0.22-1.55)	.29	0.78 (0.40-1.50)	.45	0.80 (0.47-1.34)	.39	0.91 (0.39-2.10)	.82
Subtype								
HR+/HER2-	Ref.		Ref.		Ref.		Ref.	
HER2+	1.77 (0.35-8.83)	.49	0.46 (0.11-1.99)	.30	0.81 (0.31-2.13)	.67	0.85 (0.18-4.01)	.83
TNBC	2.10 (0.66-6.70)	.21	0.88 (0.43-1.80)	.72	1.25 (0.69-2.27)	.45	1.68 (0.64-4.38)	.29
CTx								
Not performed	Ref.		Ref.		Ref.		Ref.	
Performed	0.61 (0.20-1.83)	.38	1.45 (0.59-3.59)	.42	1.02 (0.54-1.93)	.96	1.54 (0.45-5.30)	.49

LRRFS, locoregional recurrence-free survival; DRFS, Distant recurrence-free survival; RFS, recurrence-free survival; OS, overall survival; BCT, breast-conserving treatment; BSO, bilateral salpingo-oophorectomy; LN, lymph node; HG, histologic grade; HR, hormone receptor; HER2, human epidermal growth factor receptor 2; TNBC, triple negative breast cancer; CTx, chemotherapy

© 2025 Lee J et al. *JAMA Network Open*.

eTable 4. Incidence of events according to surgery type in 1:1 PS matched patients

	BCT (*n*=159)		Mastectomy (*n*=159)		*p*
Type of events	n	%	n	%	
Locoregional recurrence	8	5.1	8	5.1	.83
Distant recurrence	10	6.4	16	10.3	.18
MCBC	21	13.3	17	10.7	.13
Death	10	6.4	10	6.4	.87

PS, propensity score; BCT, breast conserving treatment; MCBC, metachronous contralateral breast cancer

© 2025 Lee J et al. *JAMA Network Open*.
