# Supplementary material for: Long-Term Oncologic Outcome of Breast-Conserving Treatment in Patients With Breast Cancer With BRCA Variants
Source: JAMA Netw Open. 2025 May 14;8(5):e259840. doi: 10.1001/jamanetworkopen.2025.9840 (PMC12079291; doi:10.1001/jamanetworkopen.2025.9840)
Supplement: Supplement 2. — Data Sharing Statement [file jamanetwopen-e259840-s002.pdf]

Data Sharing Statement

Lee. Long-Term Oncologic Outcome of Breast-Conserving Treatment in Breast Cancer With BRCA Variants. *JAMA Netw Open*. Published May 14, 2025.
doi:10.1001/jamanetworkopen.2025.9840

Data
Data available: Yes
Data types: Deidentified participant data
How to access data: chachihwan@gmail.com
When available: With publication

Supporting Documents
Document types: Informed consent form
How to access documents: chachihwan@gmail.com
When available: With publication

Additional Information
Who can access the data: Researchers whose proposed use of the data has been approved
Types of analyses: For any purpose
Mechanisms of data availability: After approval of a proposal
